# The Study of Tropical Medicine in London, Liverpool, and Edinburgh

**Published:** 1914-09-05

**Authors:** 


### THE STUDY OF TROPICAL MEDICINE

### In London, Liverpool, and Edinburgh.

At the London School of Tropical Medicine the sessions, three a year, extend over three months each. The Colonial Office requires that men selected for posts in the tropics must have attended one such session, and, in addition to showing regularity in attendance, must have passed the examination at the end of the course. Failure to do so involves loss of the appointment. Other students may take the examination or not, as they choose, and, if successful, they are entitled to a certificate to that effect. The courses are so arranged as to equip men for the Diploma in Tropical Medicine and Hygiene granted by the University of Cambridge, the Diploma in Diseases and Hygiene of the Tropics granted by the Conjoint Board, and the M.D. of the University of London (Part VI.), Tropical Medicine. The student is required to attend from 10 to 4 or 5 daily during the three months. In the wards of the hospital that adjoins the school are always to be found a number of tropical cases newly arrived in the docks. These cases afford valuable instruction, as the majority have not been under treatment prior to their arrival in the wards. The new school buildings, rendered possible by the grant from the Board of Education and the response to Mr. Chamberlain's appeal, are now complete. The main laboratory affords accommodation for sixty students; the old laboratories have been sub-divided for special purposes, among these being a course in Tropical Sanitation and Hygiene. There is resident accommodation for twenty students. Each student is provided with a bed-sitting room, and has the use of the common rooms, such as the dining-room, smoking-room, library, etc., and access to the laboratories at all times. Women graduates are received as students. Last year 178 students passed through the school.

The Liverpool School of Tropical Medicine is carried on in conjunction with the University of Liverpool, the Royal Southern Hospital, and the Royal Infirmary, for the purposes of research and clinical teaching respectively. Special wards at the hospitals have been set apart for the reception and treatment of tropical diseases, and facilities for investigation and study are given in the Johnston Laboratories of the University. The fee for a full course (lasting 13 weeks) is 13 guineas. A short course, specially arranged for advanced instruction, is also held during June (fee 4 guineas). The new laboratories will be opened early next year.

The University of Edinburgh grants a Diploma in Tropical Medicine and Hygiene. The examinations are held in December and June. The fees for the first and any subsequent appearance are: Practical bacteriology, £1 1s.; diseases of tropical climates, £1 1s.; tropical hygiene, £1 1s.; tropical clinical medicine, £1 1s.; total, £4 4s.

The University also grants a Diploma in Psychiatry. The fees for the first and second examinations are £5 5s. each. The first examination comprises anatomy, physiology, pathology, and bacteriology; the second examination, psychology (including experimental psychology), clinical neurology and psychiatry (systematic and clinical). The examinations will be held in March and June.

